# Ginsenoside Rg1 Inhibits STAT3 Expression by miR-15b-5p to Attenuate Lung Injury in Mice with Type 2 Diabetes Mellitus-Associated Pulmonary Tuberculosis

**DOI:** 10.1155/2022/9017021

**Published:** 2022-10-04

**Authors:** Tingxi Ma, Xiaohui Mao, Xiangguo Meng, Qinghu Wang

**Affiliations:** Department of Tuberculosis Comorbidity, Xi'an Chest Hospital, Xi'an, Shaanxi 710061, China

## Abstract

Type 2 diabetes mellitus (T2DM) has been regarded as a critical risk factor for pulmonary tuberculosis (PTB). Ginsenoside Rg1 has been identified as a potential therapeutic agent for T2DM by suppressing the inflammatory response. However, the effect of Rg1 on T2DM-associated PTB has not been reported. In this study, we aimed to explore the function of Rg1 in the regulation of T2DM-associated PTB. We established a T2DM-associated PTB mouse model and found that the fibrosis of lung tissues was inhibited by Rg1 in T2DM-associated PTB mice. The lung injury of T2DM-associated PTB mice was repressed by Rg1. Moreover, the levels of IL-6, TNF-*α*, and IL-1*β* in the lung tissues and serum were decreased by Rg1 in T2DM-associated PTB mice. The treatment with Rg1 inhibited the levels of free fatty acid and enhanced the expression of miR-15b-5p in lung tissues of T2DM-associated PTB mice. MiR-15b-5p targeted and inhibited the STAT3 expression. The expression of STAT3 was downregulated by Rg1, while the inhibition of miR-15b-5p reversed the downregulation. The expression of miR-15b-5p was reduced, but the expression of STAT3 was upregulated in the lung tissues of T2DM-associated PTB mice. We validated that miR-15b-5p attenuated inflammation and lung injury in the T2DM-associated PTB mouse model. The overexpression of STAT3 or the suppression of miR-15b-5p restored lung fibrosis and injury inhibited by Rg1 in T2DM-associated PTB mice. Meanwhile, the Rg1-repressed levels of IL-6, TNF-*α*, and IL-1*β* were enhanced by the overexpression of STAT3 or the suppression of miR-15b-5p. In addition, the levels of free fatty acid repressed by Rg1 were reversed by STAT3 overexpression and miR-15b-5p inhibition. Thus, we conclude that ginsenoside Rg1 inhibits the STAT3 expression by miR-15b-5p to attenuate lung injury in mice with type 2 diabetes mellitus-associated pulmonary tuberculosis.

## 1. Introduction

Type 2 diabetes mellitus (T2DM) has been regarded as a critical risk factor for pulmonary tuberculosis (PTB) [1]. Individuals with T2DM show two to eight times the risk of developing PTB [2]. Nowadays, the emerging T2DM pandemic presents a major challenge in global public health, which poses T2DM and PTB as major health issues, especially in areas where PTB is rampant [3]. Pathologically, infection caused by mycobacterium tuberculosis (MTB) is the major factor of PTB [4, 5]. The current first-line therapy for PTB includes isoniazid, rifampicin, ethambutol, and pyrazinamide and lasts for more than 6 months [6, 7]. T2DM can not only increase the susceptibility to MTB but also affect the progression of PTB and the unresponsiveness to therapies, as well as the risk of death and relapse [8, 9]. Therefore, the development of novel therapeutic strategies for T2DM-associated PTB is imperative.

Traditional Chinese medicine has been widely applied in the therapy and prevention of various diseases [10, 11]. Ginseng is one of the most widely used and studied traditional Chinese medicines worldwide, owing to its diverse pharmacological functions [11]. Among the bioactive components of ginseng, ginsenoside Rg1 (Rg1) is extensively studied [12–14]. Treatment with Rg1 increases the expression of VEGF and promotes the proliferation of endothelial cells and angiogenesis *in vitro* and *in vivo* in ischemic models [15]. In glucose-induced hepatic gluconeogenesis, Rg1 elevated phosphorylation of AKT and decreased the level of glucose [16]. A previous study indicated that Rg1 is a potential therapeutic agent for T2DM which works by suppressing inflammatory responses [17]. Nevertheless, the function of Rg1 in T2DM-associated PTB has not been reported.

Signal transducer and activator of transcription-3 (STAT3) is an important transcriptional factor of JAK signaling and plays critical roles in regulating proliferation, differentiation, apoptosis, inflammatory response, and immune reaction [18–20]. STAT3 participates in the expression and function of cytokine receptors, such as *e*interleukin-6 (IL-6), interferon-*γ* (IFN-*γ*), and platelet-derived growth factor (PDGF) [20]. Moreover, STAT3 is involved in the replication and pathogenesis of viruses and causes the adverse effects of viral infections [21]. However, studies have indicated the role of STAT3 as a mediator in MTB infection [22, 23].

MicroRNAs (miRNAs) are a form of noncoding RNAs with lengths of 20 nucleotides, which modulate target miRNA stability and transcription and consequently participate in various cellular processes including inflammation and immune responses [24]. MiR-15b-5p is a recently reported regulator of disease progression. Studies have suggested that miR-15b-5p participates in the regulation of cell proliferation, metastasis, autophagy, and other cell processes during the development of cancers and cardiovascular diseases [25–27]. A recent work also pointed out that the abnormal level of miR-15b-5p in urine may be an indicator of kidney dysfunction [28]. Yet, no studies have currently highlighted the role of miR-15b-5p in T2DM-associated PTB.

In this study, we determined the therapeutic effects of Rg1 against T2DM-associated PTB using the *in vivo* mouse model and further explored the regulatory mechanisms involving the miR-15b-5p/STAT3 axis. Our work presented Rg1 as a novel and effective treatment agent for T2DM-associated PTB.

## 2. Materials and Methods

### 2.1. Mouse Model

We first established the T2DM mouse model induced by streptozotocin (STZ). In short, C57BL/6J mice aged 4 weeks were purchased from Vital River Laboratory (Beijing, China). The STZ (60 mg/kg) was intraperitoneally injected for 5 consecutive days. Then, the blood glucose level was checked once a week after the STZ injection. A value over 16.7 mM was regarded as the successful establishment of a T2DM mouse model.

One month after STZ injection, the T2DM mice were treated with MTB. H37Rv (ATCC, USA) for PTB induction [29, 30]. The H37Rv was diluted in PBS to 2 × 10^6^ colony‐forming units (CFU)/mL, and then it was atomized by an atomizer. T2DM mice were exposed to the bacterium for 15 minutes. Rg1 was brought from Solarbio (China) and was dissolved in DMSO for stock. One month after H37Rv infection, Rg1 (2 mg/ml) and oligonucleotides (10 nmol) were administrated two times every 10 days via intraperitoneal injection within one month. PTB mice in the control group were treated with PBS. After treatment, the mice were sacrificed and lung tissues and blood were collected. To check CFU in the lung, the tissues were homogenized and incubated on agar plates at 37°C with 5% CO_2_ for 20 days. All animal experiments complied with the ARRIVE guidelines and were carried out in accordance with the National Institutes of Health Guide for the Care and Use of Laboratory Animals (NIH Publication No. 8023, revised 1978). The animal experiments in this work were approved by the animal care and use committee at the Xi'an Chest Hospital.

### 2.2. Serum Analysis

The blood was centrifuged so as to collect serum, and then the level of free fatty acid (FFA) and triglyceride (TG) were measured by a commercial detection kit (Solarbio, China) following the manufacturer's protocols.

### 2.3. Enzyme-Linked Immuno Sorbent Assay (ELISA)

The levels of IL-6, IL-1*β,* and TNF-*α* in lung tissues were measured by commercial ELISA kits and by microplate reader.

### 2.4. Histopathologic Analysis

Lung tissues were fixed in 4% paraformaldehyde (PFA), embedded with paraffin, and made into 4 *μ*m slices. The tissue damage and fibrosis were checked by haematoxylin and eosin (HE) staining and Masson's trichrome staining (Beyotime, China). For HE staining, samples were deparaffinated, rehydrated, and stained with haematoxylin and eosin. For Masson's trichrome staining, the samples were stained with haematoxylin, ferric oxide, and acid fuchsin following the commercial kit's instructions.

### 2.5. Cell Culture and Treatment

The human kidney cell line HEK293T was brought from Shanghai Cell Bank of Chinese Science Academy, maintained in CMEM (Hyclone, USA) with 10% FBS (Gibco, USA) and 1% penicillin/streptomycin. The STAT3 overexpressing vectors, siSTAT3, miR-15b-5p mimics, and miR-15b-5p inhibitors, and corresponding negative controls were synthesized by RiboBio (China). Cell transfection was performed by mixing 50 nM of oligonucleotides using Lipofectamine 2000 (Invitrogen, USA) as per the manufacturer's protocol.

### 2.6. Real-Time PCR Analysis

The lung tissues were homogenized, and total RNA was extracted by using a Trizol reagent (Thermo, USA). The cDNA was synthesized from RNA by using a PromeScript RT kit (Takara, Japan) and quantified by SYBR Green following the 2-ΔΔCt method. The RNA and miRNA expression was normalized to GAPDH and U6, respectively.

### 2.7. Western Blotting

The tissues and cells were homogenized by using RIPA buffer that contains antiprotease mixture (Thermo, USA) to obtain total protein. The protein samples were quantified with a BCA kit (Beyotime, China), resolved in SDS-PAGE, shifted into NC membranes, blocked with 5% skim milk at room temperature for 1 hour, and hatched with primary antibodies against STAT3, phosphorylated STAT3, and *β*-actin (Proteintech, China, 1 : 500) at 4°C overnight. The next day, the membranes were incubated with HRP-conjugated, antimouse, or antirabbit secondary antibodies, visualized by an ECL substrate (Millipore, USA) on a gel image system (Bio-Rad, USA).

### 2.8. Luciferase Reporter Gene Assay

The STAT3 3′UTR promoter reporter vectors that contain either wild-type or mutated miR-25b-5p binding site were cotransfected with pRL-SV40 Renilla vectors into the cell and incubated for 48 hours. The cells were then lysed, and luciferase activity was checked by a dual-luciferase reporter assay system (Promega, USA).

### 2.9. Statistics

Data were shown as mean ± standard deviation (SD) and analyzed by using SPSS 20.0 (USA). The student's *t*-test and one-way analysis of variance (ANOVA) were performed to determine a comparison between the two groups and among multiple groups. Values of *p* < 0.05 were regarded as statistically significant.

## 3. Results

### 3.1. MiR-15b-5p Expression Is Reduced and STAT3 Expression Is Increased in the T2DM-Associated PTB Mouse Model

**Figure 1:**
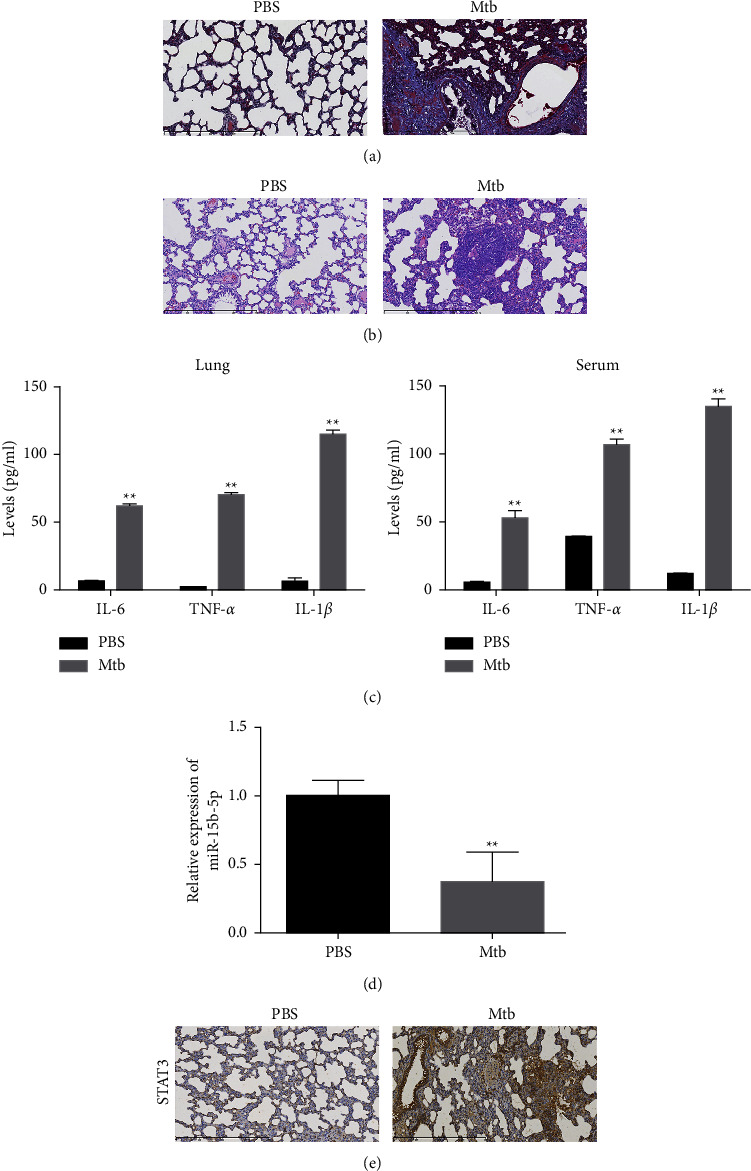
MiR-15b-5p expression is reduced and STAT3 expression is increased in the T2DM-associated PTB mouse model. (a–e) The T2DM-associated PTB mouse model was established. (a) The fibrosis of lung tissues was detected by Masson staining. (b) The lung injury was analyzed by H&E staining. (c) The levels of inflammation factors in lung tissues and serum were detected by ELISA. (d) The expression of miR-15b-5p in the lung tissues was measured by qPCR. (e) The levels of STAT3 in lung tissues were determined by IHC staining. PBS represents control group; MTb represents the MTb-induced PTB mouse group; ^*∗∗*^*p* < 0.05 vs PBS group.

We established a T2DM-associated PTB mouse model and assessed the expression of miR-15b-5p and STAT3. The fibrosis of lung tissues was observed in the T2DM-associated PTB mice (Figure 1(a)), and we observed the lung injury of T2DM-associated PTB mice (Figure 1(b)). Meanwhile, the enhanced levels of IL-6, TNF-*α*, and IL-1*β* in the lung tissues and the serum of T2DM-associated PTB mice compared with the control (PBS) group were validated (Figure 1(c)). Significantly, the expression of miR-15b-5p was reduced but STAT3 expression was upregulated in the lung tissues of T2DM-associated PTB mice, compared with the PBS group (Figures 1(d) and 1(e)).

### 3.2. Rg1 Attenuates Inflammation and Lung Injury in the T2DM-Associated PTB Mouse Model

**Figure 2:**
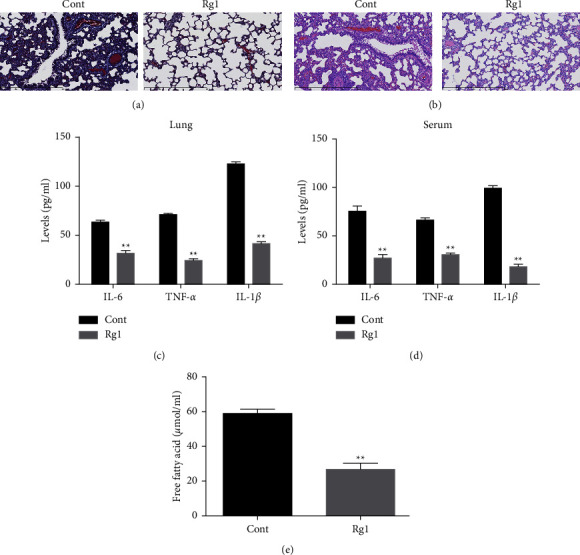
Rg1 attenuates inflammation and lung injury in T2DM-associated PTB mouse model. (a–e) The T2DM-associated PTB mice were treated with Rg1 or PBS as control. (a) The fibrosis of lung tissues was detected by Masson staining. (b) The lung injury was analyzed by H&E staining (c, d). The levels of inflammation factors in lung tissues and serum were detected by ELISA. (e) The levels of free fatty acid in lung tissues were analyzed. Cont represents the PBS treatment group; Rg1 represents the Rg1 treatment group; ^*∗∗*^*p* < 0.05 vs Cont group.

We were then interested in the effect of Rg1 on the inflammation and lung injury of T2DM-associated PTB mice. Interestingly, we observed that the fibrosis of lung tissues was inhibited by Rg1 treatment in T2DM-associated PTB mice, compared with the ones treated with PBS as control (Figure 2(a)). Consistently, the lung injury of T2DM-associated PTB mice was repressed by Rg1 (Figure 2(b)). Moreover, the levels of IL-6, TNF-*α*, and IL-1*β* in the lung tissues and serum were decreased by Rg1 treatment in T2DM-associated PTB mice, compared with the control group (Figures 2(c) and 2(d)). Moreover, the treatment with Rg1 inhibited the levels of free fatty acid (Figure 2(e)).

### 3.3. Rg1 Inhibits STAT3 Expression by Upregulating miR-15b-5p

**Figure 3:**
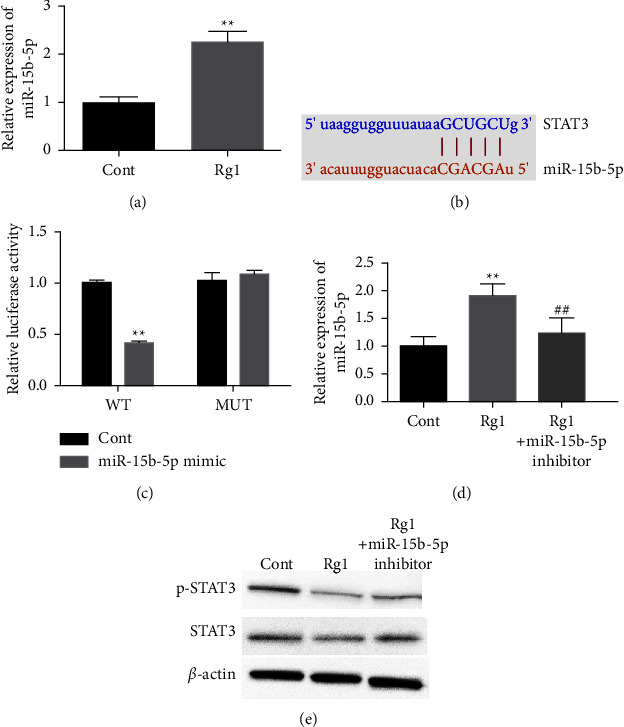
Rg1 inhibits STAT3 expression by upregulating miR-15b-5p. (a) The expression of miR-15b-5p was tested by qPCR in lung tissues of T2DM-associated PTB mice. (b) The interaction site of miR-15b-5p and STAT3 was predicted. (c, d) The 293T cells were treated with miR-15b-5p mimic. (c) The luciferase activity of STAT3 3′UTR was analyzed. (d) The expression of STAT3 was analyzed by qPCR. (e) The expression of STAT3 was detected by Western blot in 293T cells treated with Rg1 and miR-15b-5p inhibitor. Cont represents the PBS treatment group; Rg1 represents the Rg1 treatment group; Rg1 + miR-15b-5p inhibitor represents the treatment with Rg1 and miR-15b-5p inhibitor; WT represents the wild-type vectors; and MUT represents the mutated vectors; ^*∗∗*^*p* < 0.05 vs Cont group, ^##^*p* < 0.05 vs Rg1 group.

Regarding the mechanism, we found that the treatment with Rg1 enhanced the expression of miR-15b-5p in lung tissues of T2DM-associated PTB mice (Figure 3(a)). Next, the potential interaction site between miR-15b-5p and STAT3 was identified (Figure 3(b)). The luciferase activity of STAT3 3′UTR was repressed by miR-15b-5p (Figure 3(c)), and the treatment of miR-15b-5p mimic reduced the expression of STAT3 (Figure 3(d)). The expression of STAT3 was downregulated by Rg1, whereas the inhibition of miR-15b-5p reversed the downregulation (Figure 3(e)).

### 3.4. MiR-15b-5p Relieves Inflammation and Lung Injury in the T2DM-Associated PTB Mouse Model

We then validated the function of miR-15b-5p in T2DM-associated PTB mice and confirmed the upregulation of miR-15b-5p by miR-15b-5p mimic in lung tissues of T2DM-associated PTB mice (Figure S1(a)). We found that the fibrosis of lung tissues was attenuated by miR-15b-5p in T2DM-associated PTB mice (Figure S1(b)). The lung injury of T2DM-associated PTB mice was inhibited by miR-15b-5p (Figure S1(c)). Moreover, the levels of IL-6, TNF-*α*, and IL-1*β* in the lung tissues and serum were downregulated by miR-15b-5p in T2DM-associated PTB mice (Figure S1(d)). In addition, the treatment with miR-15b-5p inhibited the levels of free fatty acid (Figure S1(e)).

### 3.5. MiR-15b-5p Relieves Inflammation and Lung Injury in the T2DM-Associated PTB Mouse Model by Targeting STAT3

We found that the fibrosis of lung tissues was promoted by miR-15b-5p inhibitor compared with the control group, while the depletion of STAT3 reversed the effect in T2DM-associated PTB mice (Figure S2(a)). The lung injury of T2DM-associated PTB mice was induced by miR-15b-5p inhibitor and STAT3 knockdown inhibited the phenotype in T2DM-associated PTB mice (Figure S2(b)). Moreover, the levels of IL-6, TNF-*α*, and IL-1*β* in the lung tissues and serum were enhanced by miR-15b-5p inhibitor, compared with the control group, but the STAT3 depletion reversed the effect in T2DM-associated PTB mice (Figures S2(c) and S2(d)). Besides, the treatment with miR-15b-5p inhibitor upregulated the levels of free fatty acid, whereas the silencing of STAT3 reversed the effect (Figure S2(e)).

### 3.6. Rg1 Inhibits Inflammation and Lung Injury in the T2DM-Associated PTB Mouse Model by miR-15b-5p/STAT3 Axis

**Figure 4:**
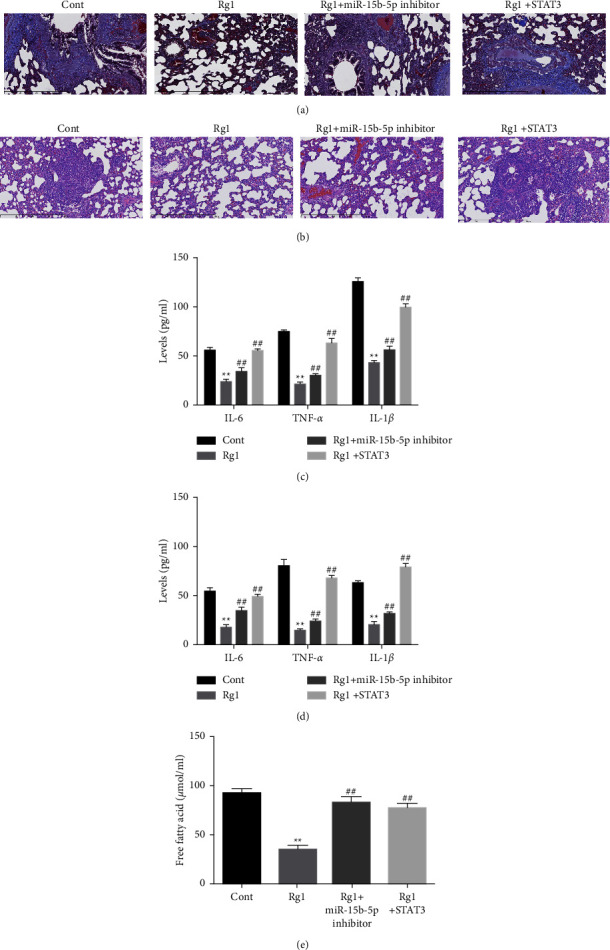
Rg1 inhibits inflammation and lung injury in the T2DM-associated PTB mouse model by miR-15b-5p/STAT3 axis. (a–e) The T2DM-associated PTB mice were treated with Rg1 and miR-15b-5p inhibitor or STAT3 overexpressing plasmid. (a) The fibrosis of lung tissues was detected by Masson staining. (b) The lung injury was analyzed by H&E staining (c, d). The levels of inflammation factors were detected by ELISA in lung tissues and serum. (e) The levels of free fatty acid were analyzed. Cont represents PTB mice treated with PBS as control; Rg1 represents the Rg1 treatment group; Rg1 + miR-15b-5p inhibitor represents the treatment with Rg1 and miR-15b-5p inhibitor; Rg1 + STAT3 represents the treatment with Rg1 and the overexpression of STAT3; ^*∗∗*^*p* < 0.05 vs Cont group, ^##^*p* < 0.05 vs Rg1 group.

Next, we observed that the overexpression of STAT3 or the suppression of miR-15b-5p restored lung fibrosis and injury inhibited by Rg1 in T2DM-associated PTB mice (Figures 4(a) and 4(b)). Meanwhile, the Rg1-repressed levels of IL-6, TNF-*α*, and IL-1*β* were enhanced by the overexpression of STAT3 or by the suppression of miR-15b-5p (Figures 4(c) and 4(d)). In addition, the levels of free fatty acid repressed by Rg1 were reversed by STAT3 overexpression and miR-15b-5p inhibition (Figure 4(e)).

## 4. Discussion

T2DM has been regarded as a critical risk factor for PTB [31, 32]. Ginsenoside Rg1 has been identified as a potential therapeutic agent for T2DM that works by suppressing inflammatory response [33]. But the effect of Rg1 on T2DM-associated PTB has not been reported. In the current world, we uncovered the function of Rg1 in the inhibition of T2DM-associated PTB, and we identified that the fibrosis of lung tissues was inhibited by Rg1 in T2DM-associated PTB mice. The lung injury of T2DM-associated PTB mice was repressed by Rg1.

Rg1 has presented multiple biomedical activities in several diseases. It has been reported that ginsenoside Rg1 enhances cerebral angiogenesis in ischemic mice by the PI3K/Akt/mTOR signaling [15]. Ginsenoside Rg1 protects PTSD-like behaviors by inducing synaptic proteins, as well as by repressing Kir4.1 and TNF-*α* in the hippocampus [34]. Ginsenoside Rg1 attenuates ischemic/reperfusion-induced neuronal injury by the miR-144-Nrf2-ARE signaling [35]. Ginsenoside Rg1 inhibits cardiomyocyte inflammation and apoptosis by the TLR4/NF-kB/NLRP3 signaling [12]. Ginsenoside Rg1 suppresses glucagon-associated hepatic gluconeogenesis by the interaction of Akt-FoxO1 [16]. Consistent with the previous studies, we identified that the levels of inflammatory factors, IL-6, TNF-*α*, and IL-1*β*, in the lung tissues and serum were decreased by Rg1 in T2DM-associated PTB mice. Moreover, the treatment with Rg1 inhibited the levels of free fatty acid. These data elucidate the critical impact of Rg1 on T2DM-associated PTB, enriching the understanding of the function of natural compounds in the treatment of T2DM-associated PTB.

Furthermore, it has been reported that the phosphorylation of STAT3 represses the microRNA-19b expression to promote lung injury in T2DM-associated PTB [29]. The enhancement of miR-196b-5p inhibits the uptake of BCG by regulating SOCS3 and promoting STAT3 in PTB [36]. These studies indicated the participation of STAT3 in PTB. Our data revealed that the treatment with Rg1 enhanced the expression of miR-15b-5p and suppressed the expression of STAT3 in lung tissues of T2DM-associated PTB mice. Further mechanical research demonstrated that miR-15b-5p targeted and inhibited the STAT3 expression, and the inhibition of miR-15b-5p reversed the Rg1-downregulated STAT3 level. Besides, the expression of miR-15b-5p was reduced but STAT3 expression was upregulated in the lung tissues of T2DM-associated PTB mice. These findings suggested that Rg1 may upregulate the miR-15b-5p to suppress STAT3 expression and function in the PTB model.

Furthermore, we validated that miR-15b-5p relieves inflammation and lung injury in the T2DM-associated PTB mouse model. The overexpression of STAT3 or the suppression of miR-15b-5p restored lung fibrosis and injury inhibited by Rg1 in T2DM-associated PTB mice. Meanwhile, the Rg1-repressed levels of IL-6, TNF-*α*, and IL-1*β* were enhanced by the overexpression of STAT3 or by the suppression of miR-15b-5p. In addition, the level of free fatty acid repressed by Rg1 was reversed by STAT3 overexpression and miR-15b-5p inhibition. These data imply that Rg1 may affect T2DM-associated PTB by targeting the miR-15b-5p/STAT3 signaling. However, the regulatory mechanisms underlying Rg1-upregulated miR-15b-5p expression still need further exploration and this regulatory axis should be verified in *in vitro* cell models. Other potential targets and mechanisms of Rg1 in the treatment of T2DM-associated PTB should be explored by more studies.

In conclusion, we can say that ginsenoside Rg1 inhibits STAT3 expression by miR-15b-5p to attenuate lung injury in mice with type 2 diabetes mellitus-associated pulmonary tuberculosis.

## Data Availability

No data were used in this study.

## Supplementary Materials

Supplementary Materials Figure S1. MiR-15b-5p relieves inflammation and lung injury in the T2DM-associated PTB mouse model. (A–E) The T2DM-associated PTB mice were treated with miR-15b-5p mimic. (A) The expression of miR-15b-5p in lung tissues was analyzed by qPCR. (B) The fibrosis of lung tissues was detected by Masson staining. (C) The lung injury was analyzed by H&E staining. (D) The levels of inflammation factors were detected by ELISA in lung tissues and serum. (E) The levels of free fatty acid were analyzed. Cont represents PTB mice treated with PBS as control and miR-15b-5p mimic represents the treatment with miR-15b-5p mimics; ^*∗∗*^*p* < 0.05 vs Cont group. Figure S2. MiR-15b-5p relieves inflammation and lung injury in the T2DM-associated PTB mouse model by targeting STAT3. (A–E) The T2DM-associated PTB mice were treated with miR-15b-5p inhibitor and STAT3 siRNA. (A) The fibrosis of lung tissues was detected by Masson staining. (B) The lung injury was analyzed by H&E staining. (C, D) The levels of inflammation factors were detected by ELISA in lung tissues and serum. (E) The levels of free fatty acid were analyzed. Cont represents PTB mice treated with PBS as control; miR-15b-5p inhibitor represents the treatment with miR-15b-5p inhibitor; and miR-15b-5p inhibitor + siSTAT3 represents the treatment with miR-15b-5p inhibitor and the depletion of STAT3; ^*∗∗*^*p* < 0.05 vs Cont group; ^##^*p* < 0.05 vs miR-15b-5p inhibitor group.

## References

[1] Al-Rifai R. H., Pearson F., Critchley J. A., Abu-Raddad L. J. (2017). Association between diabetes mellitus and active tuberculosis: a systematic review and meta-analysis. *PLoS One*.

[2] Ayelign B., Negash M., Genetu M., Wondmagegn T., Shibabaw T. (2019). Immunological impacts of diabetes on the susceptibility of Mycobacterium tuberculosis. *Journal of Immunology Research*.

[3] Workneh M. H., Bjune G. A., Yimer S. A. (2017). Prevalence and associated factors of tuberculosis and diabetes mellitus comorbidity: a systematic review. *PLoS One*.

[4] Dheda K., Barry C. E., Maartens G. (2016). Tuberculosis. *The Lancet*.

[5] Furin J., Cox H., Pai M. (2019). Tuberculosis. *The Lancet*.

[6] Suárez I., Fünger S. M., Kröger S., Rademacher J., Fätkenheuer G., Rybniker J. (2019). The diagnosis and treatment of tuberculosis. *Deutsches Arzteblatt International*.

[7] Churchyard G., Kim P., Shah N. S. (2017). What we know about tuberculosis transmission: an overview. *The Journal of Infectious Diseases*.

[8] Martinez N., Kornfeld H. (2014). Diabetes and immunity to tuberculosis. *European Journal of Immunology*.

[9] Dooley K. E., Chaisson R. E. (2009). Tuberculosis and diabetes mellitus: convergence of two epidemics. *The Lancet Infectious Diseases*.

[10] Li Y., Wang L., Wang P. (2020). Ginsenoside-Rg1 rescues stress-induceddepression-like behaviors via suppression of oxidative stress and neural inflammation in rats. *Oxidative Medicine and Cellular Longevity*.

[11] Jiang N., Lv J., Wang H. (2020). Ginsenoside Rg1 ameliorates chronic social defeat stress-induceddepressive-like behaviors and hippocampal neuroinflammation. *Life Sciences*.

[12] Luo M., Yan D., Sun Q. (2020). Ginsenoside Rg1 attenuates cardiomyocyte apoptosis and inflammation via the TLR4/NF-kB/NLRP3 pathway. *Journal of Cellular Biochemistry*.

[13] Huang L., Cai H. A., Zhang M. S., Liao R. Y., Huang X., Hu F. D. (2021). Ginsenoside Rg1 promoted the wound healing in diabetic foot ulcers via miR-489-3p/Sirt1 axis. *Journal of Pharmacological Sciences*.

[14] Wang Z., Wang L., Jiang R. (2021). Ginsenoside Rg1 prevents bone marrow mesenchymal stem cell senescence via NRF2 and PI3K/Akt signaling. *Free Radical Biology and Medicine*.

[15] Chen J., Zhang X., Liu X. (2019). Ginsenoside Rg1 promotes cerebral angiogenesis via the PI3K/Akt/mTOR signaling pathway in ischemic mice. *European Journal of Pharmacology*.

[16] Liu Q., Zhang F. G., Zhang W. S. (2017). Ginsenoside Rg1 inhibits glucagon-induced hepatic gluconeogenesis through akt-FoxO1 interaction. *Theranostics*.

[17] Alolga R. N., Nuer-Allornuvor G. F., Kuugbee E. D., Yin X., Ma G. (2020). Ginsenoside Rg1 and the control of inflammation implications for the therapy of type 2 diabetes: a review of scientific findings and call for further research. *Pharmacological Research*.

[18] Fan Y., Mao R., Yang J. (2013). NF-*κ*B and STAT3 signaling pathways collaboratively link inflammation to cancer. *Protein Cell*.

[19] Hillmer E. J., Zhang H., Li H. S., Watowich S. S. (2016). STAT3 signaling in immunity. *Cytokine & Growth Factor Reviews*.

[20] Yu H., Pardoll D., Jove R. (2009). STATs in cancer inflammation and immunity: a leading role for STAT3. *Nature Reviews Cancer*.

[21] Queval C. J., Song O. R., Deboosère N. (2016). STAT3 represses nitric oxide synthesis in human macrophages upon mycobacterium tuberculosis infection. *Scientific Reports*.

[22] Li M., Jiao L., Lyu M. (2020). Association of IL27 and STAT3 genetic polymorphism on the susceptibility of tuberculosis in western Chinese Han population. *Infection, Genetics and Evolution*.

[23] Rottenberg M. E., Carow B. (2014). SOCS3 and STAT3, major controllers of the outcome of infection with mycobacterium tuberculosis. *Seminars in Immunology*.

[24] Lu T. X., Rothenberg M. E. (2018). MicroRNA. *The Journal of Allergy and Clinical Immunology*.

[25] Dong Y., Zhang N., Zhao S., Chen X., Li F., Tao X. (2019). miR-221-3p and miR-15b-5p promote cell proliferation and invasion by targeting Axin2 in liver cancer. *Oncology Letters*.

[26] Wu B., Liu G., Jin Y. (2020). miR-15b-5p promotes growth and metastasis in breast cancer by targeting HPSE2. *Frontiers Oncology*.

[27] Zhu Y., Yang T., Duan J., Mu N., Zhang T. (2019). MALAT1/miR-15b-5p/MAPK1 mediates endothelial progenitor cells autophagy and affects coronary atherosclerotic heart disease via mTOR signaling pathway. *Aging (Albany NY)*.

[28] Prabu P., Rome S., Sathishkumar C. (2019). MicroRNAs from urinary extracellular vesicles are non-invasive early biomarkers of diabetic nephropathy in type 2 diabetes patients with the ‘Asian Indian phenotype’. *Diabetes and Metabolism*.

[29] Wang X., Lin Y., Liang Y. (2020). Phosphorylated STAT3 suppresses microRNA-19b/1281 to aggravate lung injury in mice with type 2 diabetes mellitus-associated pulmonary tuberculosis. *Journal of Cellular and Molecular Medicine*.

[30] Liu F., Chen J., Wang P. (2018). MicroRNA-27a controls the intracellular survival of Mycobacterium tuberculosis by regulating calcium-associated autophagy. *Nature Communications*.

[31] Xia L. L., Li S. F., Shao K., Zhang X., Huang S. (2018). The correlation between CT features and glycosylated hemoglobin level in patients with T2DM complicated with primary pulmonary tuberculosis. *Infection and Drug Resistance*.

[32] Yang W. B., Wang H. L., Mao J. T. (2020). The correlation between CT features and insulin resistance levels in patients with T2DM complicated with primary pulmonary tuberculosis. *Journal of Cellular Physiology*.

[33] Lee H. M., Lee O. H., Kim K. J., Lee B. Y. (2012). Ginsenoside Rg1 promotes glucose uptake through activated AMPK pathway in insulin-resistant muscle cells. *Phytotherapy Research*.

[34] Zhang Z., Song Z., Shen F. (2021). Ginsenoside Rg1 prevents PTSD-like behaviors in mice through promoting synaptic proteins, reducing Kir4.1 and TNF-alpha in the Hippocampus. *Molecular Neurobiology*.

[35] Chu S. F., Zhang Z., Zhou X. (2019). Ginsenoside Rg1 protects against ischemic/reperfusion-induced neuronal injury through miR-144/Nrf2/ARE pathway. *Acta Pharmacologica Sinica*.

[36] Yuan Y., Lin D., Feng L. (2018). Upregulation of miR-196b-5p attenuates BCG uptake via targeting SOCS3 and activating STAT3 in macrophages from patients with long-term cigarette smoking-related active pulmonary tuberculosis. *Journal of Translational Medicine*.

