# Supplementary figures and images for: Ginsenoside Rg1 Inhibits STAT3 Expression by miR-15b-5p to Attenuate Lung Injury in Mice with Type 2 Diabetes Mellitus-Associated Pulmonary Tuberculosis
Source: Evid Based Complement Alternat Med. 2022 Oct 4;2022:9017021. doi: 10.1155/2022/9017021 (PMC9553455; doi:10.1155/2022/9017021)

**
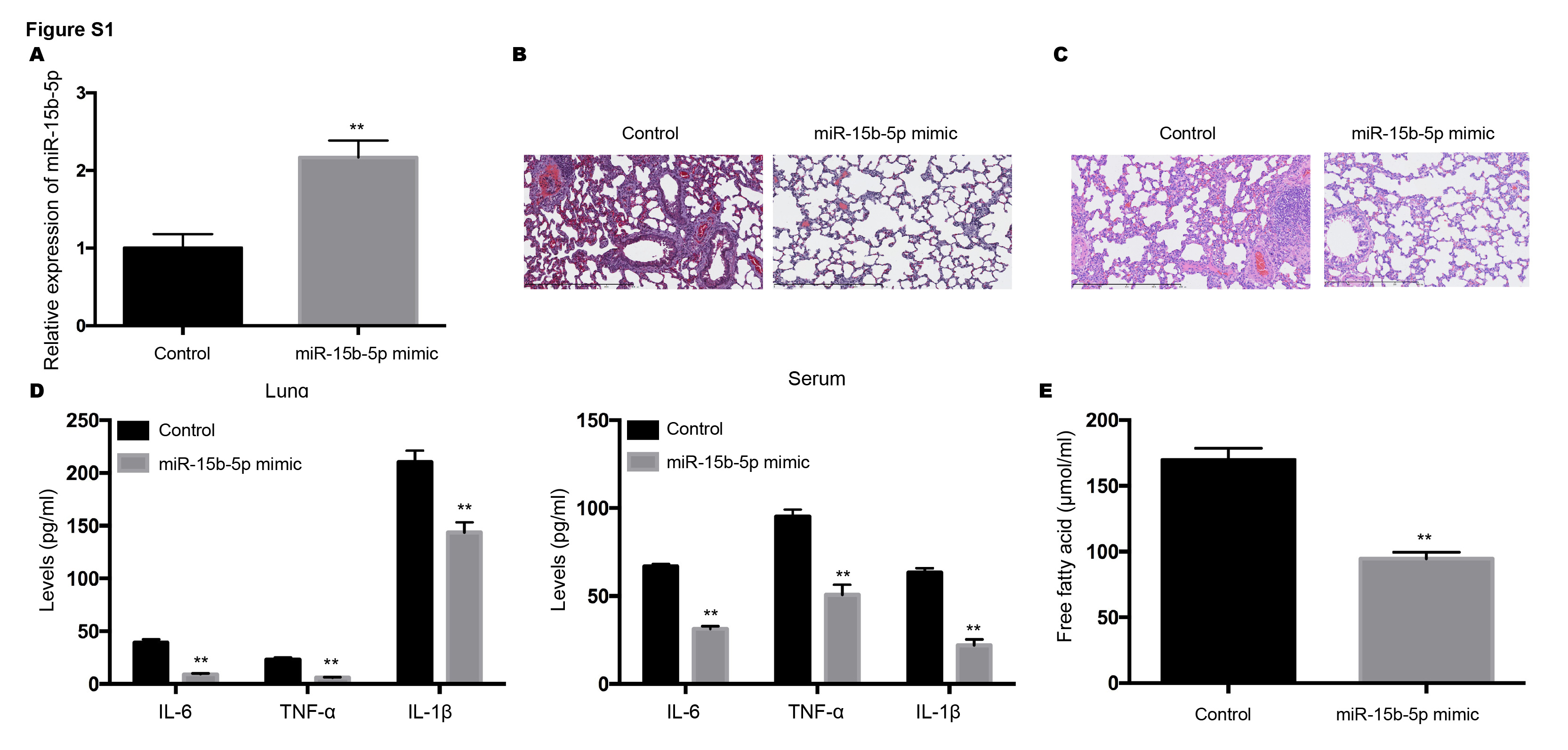
**

**
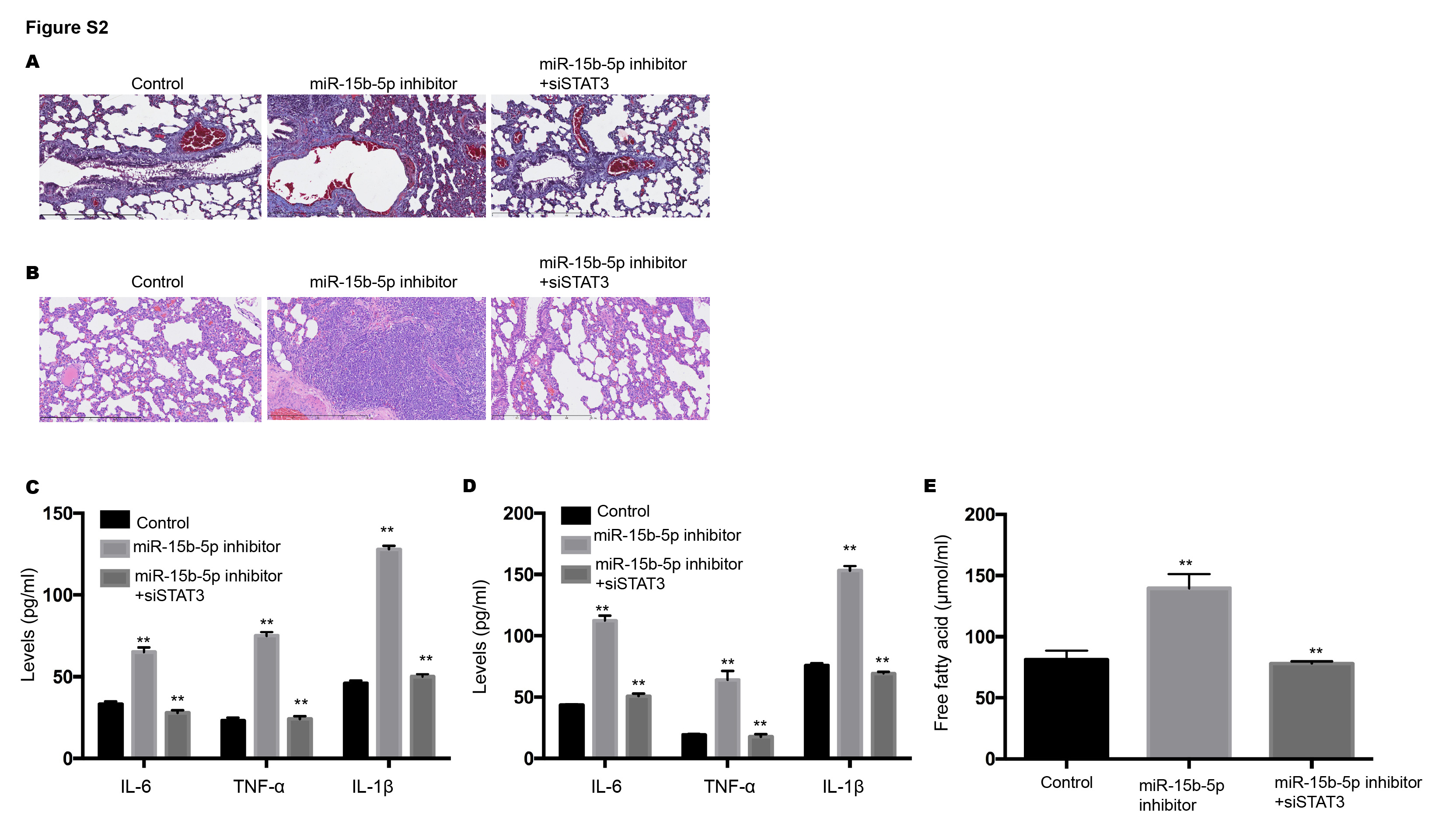
**

Supplement: Supplementary Materials — Figure S1. MiR-15b-5p relieves inflammation and lung injury in the T2DM-associated PTB mouse model. (A–E) The T2DM-associated PTB mice were treated with miR-15b-5p mimic. (A) The expression of miR-15b-5p in lung tissues was analyzed by qPCR. (B) The fibrosis of lung tissues was detected by Masson staining. (C) The lung injury was analyzed by H&E staining. (D) The levels of inflammation factors were detected by ELISA in lung tissues and serum. (E) The levels of free fatty acid were analyzed. Cont represents PTB mice treated with PBS as control and miR-15b-5p mimic represents the treatment with miR-15b-5p mimics; ∗∗p < 0.05 vs Cont group. Figure S2. MiR-15b-5p relieves inflammation and lung injury in the T2DM-associated PTB mouse model by targeting STAT3. (A–E) The T2DM-associated PTB mice were treated with miR-15b-5p inhibitor and STAT3 siRNA. (A) The fibrosis of lung tissues was detected by Masson staining. (B) The lung injury was analyzed by H&E staining. (C, D) The levels of inflammation factors were detected by ELISA in lung tissues and serum. (E) The levels of free fatty acid were analyzed. Cont represents PTB mice treated with PBS as control; miR-15b-5p inhibitor represents the treatment with miR-15b-5p inhibitor; and miR-15b-5p inhibitor + siSTAT3 represents the treatment with miR-15b-5p inhibitor and the depletion of STAT3; ∗∗p < 0.05 vs Cont group; ##p < 0.05 vs miR-15b-5p inhibitor group. [file 9017021.f1.docx]
